# Association between dental amalgam fillings and Alzheimer’s disease

**DOI:** 10.1186/s13195-016-0175-0

**Published:** 2016-01-18

**Authors:** Amin Zarghami, Seyyed Mohammad Masoud Hojjati

**Affiliations:** Department of Neurology, Ayatollah Rohani Hospital, Babol University of Medical Sciences, Babol, Iran

Sun et al. [1] evaluated the association between dental amalgam fillings and Alzheimer’s disease (AD) in a large scale Taiwanese population aged 65 years and older in a cross-sectional study and concluded that women’s exposure to amalgam was significantly associated with AD. Their findings, as the first data from a population-based study, are valuable while a previous retrospective cohort study in New Zealand could not investigate the association between dental amalgam exposure and AD due to insufficient cases [2].

In comparison with previous studies which indicated higher mercury concentrations in serum and cerebrospinal fluid samples of living and brain samples of deceased patients with Alzheimer’s disease [3, 4], Sun et al. exclusively assessed epidemiologic variables based on the Longitudinal Health Insurance Database. Several aspects of the results of their study need to be interpreted with caution due to their cross-sectional study design, and multiple limitations affected the power of study. Firstly, the duration of National Health Insurance (NHI) coverage since 2001 would include patients in their sixth and seventh decades but the pathophysiology of AD may have started much earlier. On the other hand, they mention that actual fillings were underestimated during the patients’ life-times but this fact also existed for non-AD participants because most of the dental preservation procedures were performed during their youth to middle age. Although they considered multiple face amalgam restorations based on the different NHI codes, they did not differentiate between single and multiple fillings and unfortunately there was no specific analysis of whether there is a difference between single versus multiple-surface amalgam restoration or not. On the other hand, based on their findings, what is the current interpretation for sex differences with regard to amalgam-associated AD? Are females more affected by this agent or not?

Altogether, they describe well their limitations in such an epidemiological study but, according to what they found, future investigations are needed to definitively explore the association between AD and amalgam by evaluating the duration of exposure to amalgam, considering the number of dental fillings, the size of restorations and the covering surface area and other probable risk factors that play roles in the pathophysiology of AD.

## Abbreviations

AD: Alzheimer’s disease
NHI: National Health Insurance

## References

[1] Sun Y, Nfor O, Huang J, Liaw Y (2015). Association between dental amalgam fillings and Alzheimer’s disease: a population-based cross-sectional study in Taiwan. Alzheimers Res Ther.

[2] Bates M, Fawcett J, Garrett N, Cutress T, Kjellstrom T (2004). Health effects of dental amalgam exposure: a retrospective cohort study. Int J Epidemiol.

[3] Mutter J, Naumann J, Sadaghiani C, Schneider R, Walach H (2004). Alzheimer disease: mercury as pathogenetic factor and apolipoprotein E as a moderator. Neuro Endocrinol Lett.

[4] Gerhardsson L, Blennow K, Lundh T, Londos E, Minthon L (2009). Metal concentrations in plasma and cerebrospinal fluid in patients with Alzheimer’s disease. Dement Geriatr Cogn Disord.

